# Assessment of heavy metal exposure and cancer risk in Addis Ababa: Trends, risk factors and demographic variations in urinary cadmium, lead and chromium levels

**DOI:** 10.1016/j.toxrep.2025.102122

**Published:** 2025-09-02

**Authors:** Tsigereda Assefa Alemayehu, Andualem Mekonnen Hiruy, Mehari Meles, Belay Tefera, Tadesse Alemu Terfie

**Affiliations:** a Center for Environmental Science, CNCS, Addis Ababa University, PO Box 1176, Addis Ababa, Ethiopia; b Ethiopian Public Health Institute, PO Box 1242, Addis Ababa, Ethiopia; c Faculty of Chemical and Food Engineering, Bahir Dar Institute of Technology, Bahir Dar University, Ethiopia

**Keywords:** Biomarker, Creatinine, Cancer risk, Heavy metals, Urine analysis

## Abstract

This study aims to assess the exposure levels of Pb, Cd, and Cr and evaluate trends in heavy metal exposure among the population of Addis Ababa. A cross-sectional study was conducted involving 417 participants randomly selected from the population. Spot urine and water samples, socio-demographic characteristics, and food consumption frequency were collected. Heavy metals were analyzed using microwave plasma-atomic emission spectroscopy. Metal exposure risk from drinking water was assessed. The median concentrations of urinary Pb, Cr, and Cd were 19.4865 µg/g creatinine, 55.65 µg/g creatinine, and below the detection limit (Bd), respectively. Female participants and individuals who consumed meat daily had the highest median concentration of Pb (p < 0.005). Those who drank two or more cups of water daily had lower Pb (P < 0.01). Females who consumed eggs daily and drank two or more cups of water had the highest concentration of Cd, ranking in the 75th percentile. The median Cr concentration was higher in underweight participants (BMI < 18.5 kg/m²) at 88.41 µg/g creatinine and in overweight participants (BMI ≥ 25 kg/m²) at 66.49 µg/g creatinine compared to normal-weight participants, who had a median concentration of 48.44 µg/g creatinine (P < 0.01). Three metals were detected in 14.4 % and two metals in 52.8 %, and their levels showed an increasing trend over 12 years. Health risk analysis revealed that the highest Cumulative Incremental Life Cancer Risk (CILCR) values were found in Kirkos (KR-10–1.9 ×10^−3^), Lideta (LI-10–2.33 ×10^−3^), and Nifas Silk (NS-11–2.46 ×10^−3^) districts, indicating a significant cancer risk associated with cumulative exposure.

## 1 Introduction

Heavy metals are essential materials that we encounter in various aspects of our daily lives. They are used in home appliances, cooking utensils, doors and windows, as well as in pigments for paints, car bodies and batteries [17]. Industries such as leather tanning, electroplating, metalworking and automotive services rely heavily on these metals [36]. Due to their widespread applications, heavy metals are present abundantly in the abiotic environment including air, soil, water, sediment and food sources [40]. Plants like vegetables, fruits, and other cereals that grown in contaminated soil or are irrigated with polluted water can absorb these metals [21], [51], [55]. Furthermore, ambient air is polluted with heavy metals due to emissions from automobiles, industrial activities, construction and mining [27], [39].

Humans can be exposed to heavy metals from various sources including drinking water, air, food, dental amalgam, cigarette smoking and occupational environments [44], [57]. Some heavy metals, such as Fe, Zn, Co and Cu are essential metals for proper functioning of the body but can be toxic at higher concentration [28]. When taken through food and water heavy metals like Pb, cadmium (Cd), and mercury (Hg) have no beneficial roles and are classified as toxic substances that pose significant threats to human health [43], [5]. Both acute and chronic exposure to heavy metals can damage tissues and vital organs including the liver, lungs and kidneys [33], [34], [44]. Metals such as Cd, As and Cr contribute to the formation of reactive oxygen species and leading to oxidative stress that affects various cellular components, inhibit enzymes, and cause lipid peroxidation and protein oxidation [63], [67]. Cd can accumulate in the kidneys resulting tubular injury, disrupt kidney function [45] and potential cancer risk [24], [46]. Exposure to Pb has been linked to reduced cognitive function, increased blood pressure, cardiovascular disease, Parkinson’s disease, diabetes and mortality [10], [48], [6]. Additionally, Pb interferes with the metabolism of essential elements such as Fe leading to iron deficiency [23], [32]

The global focus on preventing the harmful effects of heavy metals in both environmental and occupational settings has increased attention on the practice of biomonitoring [47]. Biomarkers such as urine, blood, nails and tissues are used to assess the burden of heavy metals from various exposure sources. For instance, in Australia, Cd was identified in the kidney cortex, lung, and liver tissues of individuals who died accidentally [54]. In, Ethiopia blood Pb levels ranging from 11.73 to 36.52 μg/L were found among garage workers [3]. Elevated Ni levels were observed in blood, urine and hair samples of cigarette smokers in Saudi Arabia [6]. Additionally, Cu and Pb were detected in an industrial area of Tanzania [37] while, Co, Mo, and Pb were found among the residents of Akaki-Kaliti sub-city in Addis Ababa, Ethiopia [66].

Despite a strong interest in understanding heavy metal exposure in the general population of Ethiopia, the country faces significant challenges in conducting bio-monitoring due to economic constraints leading to fragmented and inadequate monitoring efforts. Therefore, research on heavy metal exposure and health risk assessment in the general population of Ethiopia is limited. Some studies have raised concerns about occupational exposure in various work environments [1], [18], [3]. One study found elevated concentrations of As, B, Mo, Mn, Li, and Zn from the general population of Rift valley area [29]. However, data on Pb, Cd and Cr exposure in the general population of Addis Ababa remains scarce. The current research aimed to assess levels of Cd, Pb, and Cr exposure among the general population of Addis Ababa by using urine as biomarker, along with evaluating the health hazard and risk. It also identified trends in heavy metal levels and identified potential risk factors. The findings will provide baseline information, offer valuable insights into public health challenges related to environmental contaminants, and highlights the importance of monitoring and intervention strategies.

## 2 Methods

### 2.1 Study area

**Fig. 1:**
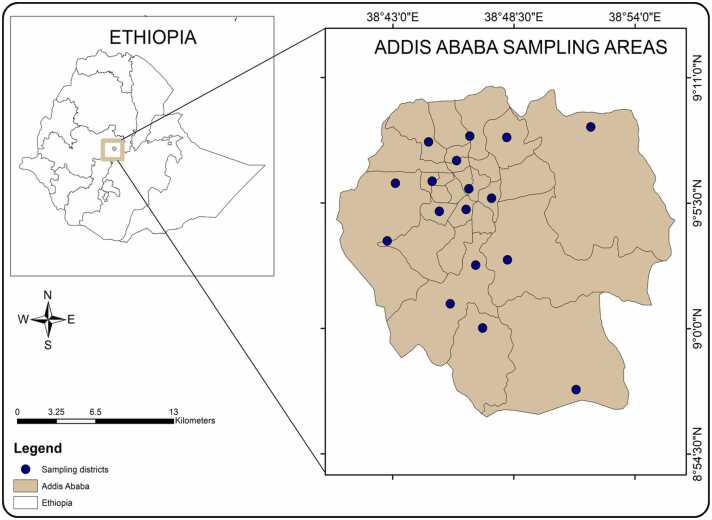
Sampling districts in Addis Ababa.

This study was conducted in Addis Ababa, the capital city of Ethiopia, the seat of the federal government and a hub of diplomacy in Africa. It is located in the central part of the country at an altitude of 2355 meters above sea level [13]. The city is positioned at a longitude of 38° 44' E and latitude of 9° 1′ N (Fig. 1). It is the largest metropolitan area covering an area of 527 square kilometers. Addis Ababa is home to a population exceeding five million (CSA, 2024). Its climate is characterized as moderate, ranging from Afro-Alpine to warm temperate, with average annual temperatures falling between 9.89 and 24.64 °C and an annual precipitation of 1874 millimeters.

### 2.2 Study population and sampling design

A cross-sectional study was conducted among the residents of Addis Ababa from May 2022 to June 2023. Enumeration areas (EAs) were identified using data from the Latest Population and Housing Census conducted by the national Central Statistics Agency (CSA) in 2019. Addis Ababa is currently divided in to 11 sub-cities and 116 districts, randomly 417 households were chosen specifically from the following districts: Bole-04 (BO-04), Bole-12 (BO-12) Addis Ketema-04 (AD-04), Arada-03 (AR-03), Akai Kality-07 (AK-07), Akai Kality-09 (AK-09), Kirkos-07 (KR-07), Kirkos-09 (KR-09), Kolfe Keranyo-01 (KO-01), Kolfe Keranyo-10 (KO-10), Lideta-01 (LI-01), Gulele-03 (GU-03), Gulele-09 (GU-09), Nifas Silk-09 (NS-09), Nifas Silk-11 (NS-11), Yeka-02 (YK-02), Yeka-12 (YK-12). The study targeted men and women aged 18 and older who had resided in Addis Ababa. One individual from each household was randomly selected for inclusion in the study. Participants under 18 years of age, those who were very ill and individuals unwilling to participate were excluded.

### 2.3 Data collection

Data collection involved conducting interviews with participants using a structured questionnaire which was administered after obtaining ethical clearance and written informed consent from each individual. The questionnaire gathered socio-demographic information, including sex, age, body weight and height and education level. It also examined factors relative to heavy metal exposure, such as occupation type and duration of residence in Addis Ababa, as well as personal habits like cigarette smoking. Additionally, the questionnaire assessed food consumption frequency for items such as vegetables, milk, eggs and meat, along with daily water intake under the assumption that these could potentially contributors to heavy metal exposure.

### 2.4 Sample collection

A total of 417 spot urine samples were collected from residents across 17 districts in Addis Ababa. Each sample was collected by using a urine specimen plastic cup that had been pre-rinsed with nitric acid and deionized water. Additionally three tap water sample were collect from each district, resulting in a total of 51 water samples. This approach was based on the assumption that each district is supplied by a single water source and that water quality is consistent throughout the district. The water samples were collected in clean, acid-washed polyethylene bottles and preserved with nitric acid. Both urine and water samples were separately stored in a cold box and transported to the Ethiopian Public Health Institute (EPHI) laboratory. The urine samples were kept at −20^0^c in a refrigerator until heavy metal analysis began.

### 2.5 Sample preparation and analysis

Urine samples for heavy metal analysis were prepared using the wet digestion method outlined by [37]. The concentrations of Cd, Pb, and Cr in both urine and water samples were quantified using Microwave Plasma-Atomic Emission Spectroscopy (Agilent 4210 MP-AES, USA) at the Food and Drug Authority laboratory. Additionally, urinary creatinine levels were measured in the Clinical Chemistry Department of EPHI using an enzymatic method [52].

### 2.6 Health risk assessment

The chronic health effects of heavy metals from tap water consumption were evaluated using estimated daily intake (EDI) (36), the target hazard quotient and (THQ), hazard index (HI) based on question 1–3 [15], [24], [4].

(1) $$\mathrm{EDI}=\frac{C*\mathrm{IR}*\mathrm{EF}*\mathrm{ED}}{\mathrm{BW}*\mathrm{AT}}$$

Where: C is the mean metal concentration in water (µg/L), IR = ingestion rate (L/day), exposure frequency (days/year), ED is the exposure duration (Years), BW is the body weight (60 kg) and AT is the average exposure period (365 days per x number of exposure years, up to 65 year).

The potential non-cancer health risks associated with heavy metals intake from water consumption were quantified using the target hazard quotient (THQ) and hazard index (HI), according to the question 2 and 3, respectively.

(2) $$\mathrm{THQ}=\frac{\mathrm{EDI}\;}{\mathrm{RfD}}$$

Where: EDI represents the estimated daily intake in L/day for an individual and RfD indicates the oral reference dose. The RfD values (mg/kg/day) for Pb, Cr (total), and Cd are 0.0014, 0.1 and 0.0035 mg/kg/day, respectively [24], [56], [61], [65].

(3) $$\mathrm{HI}=\sum_{i=1}^{n}\mathrm{THQ}=\mathrm{THQ Cd}+\mathrm{THQ Pb}+\mathrm{THQ Cr}$$

If the Hazard index (HI) is greater than 1, the exposure is likely to cause obvious adverse effects to the population.

The carcinogenic risks, the possibility of an individual to developing cancer during their entire lifetime due to exposure to heavy metals, were estimated by computing the Incremental Life Cancer Risk (ILCR) and the Cumulative Incremental Life Cancer Risk (CILCR) using 4), (5), respectively [12], [24]

Where: CSFo stands for the cancer slope factor, which represents the risk associated with carcinogenic chemicals over the lifetime. The value of CSFo for Pb, Cr and Cd was found to be 8.5 41 and 61, mg /kg/day [41].

4) ILCR=EDI*CSFo(

(5) $$\mathrm{CILCR}=\sum_{i=1}^{n}\mathrm{ILCR}=\mathrm{ILCR Cd}+\mathrm{ILCR Pb}+\mathrm{ILCR Cr}$$

### 2.7 Ethical clearance

The research proposal and application form were submitted to the Ethiopian Public Health Institute (EPHI). Ethical clearance for this study was granted by EPHI under approval No. EPHI-IRB-406–2021. The authors express their gratitude to the Research Ethics Committee of EPHI for their support.

### 2.8 Data analysis

Data analysis was conducted using IBM SPSS 20 software, calculating descriptive statistics such as, mean, median and percentiles. The 25th, 50th and 75th percentiles were used for urinary heavy metal analysis, while mean and standard deviation were applied for heavy metals in water. The normality of the data was evaluated to determine the suitability of statistical tests and was found to be non-normally distributed. A Mann-Whitney *U* test and kruskal-wallis H test were employed to examine statistical differences in the distribution of heavy metals based socio-demographic characteristics, variations among districts, frequency of food consumption (including vegetables, milk, egg, and meat) and daily water intake habits, which were gathered through an interview questionnaire.

## 3 Results and discussion

### 3.1 Results

#### 3.1.1 Urinary concentration of Pb, Cd, and Cr by districts

The urinary concentrations of Pb, and Cd were compared to the normal values established by the Centers for Disease Control and Prevention (CDC) and the Agency for Toxic Substances and Disease Registry (ATSDR). For the general population, the normal range for Pb is 16–60 µg/g creatinine while for Cd is 0.193 µg/g creatinine, respectively (Table 1). However, we were unable to find a normal value for creatinine-adjusted Cr. To assess trend in heavy metal exposure among residents of Addis Ababa, we compared our result to a previous study conducted in 2010 in Addis Ababa by Yard et al. [66].

**Table 1:** Urinary heavy metal values used to compare the result.

Metal	Unit	ATSRD	CDC	[66]
Pb	µg/g creatinine	-	16–60	0.856
Cd	µg/g creatinine	0.193	-	0.127
Cr	µg/g creatinine	-	-	0.412

The median concentration of Pb was within the CDC guideline limit for a healthy population (Table 2). However, the 75th percentile in the GU-03 and GU-09 districts exceeded the upper limit, recording levels of 68.36 and 61.33 µg/g creatinine, respectively. A significant difference (P < 0.01) in the median concentration of Pb was observed across the districts. Cd was not detected in the median, 25th and 75th percentiles in seven districts: BO-04, AD-04, AR-03, BO-12, GU-09, KO-01 and KR-10. In contrast, GU-03, LI-01 and NS-09 showed higher median concentrations above the recommended limit, measuring 0.52, 1.60 and 12.19 µg/g creatinine respectively. Additionally, elevated Cd concentrations exceeding the ATSDR limit were noted in 75th percentile across ten districts. A statistical difference was also observed across the districts (P < 0.01). Cr was detected in all districts at varying concentration. In the districts of NS-09, NS-11, LI-01, KR-07, AR-03 and AK-09, the concentration was below 50 µg/g creatinine. On the other hand, specifically higher concentration ≥ 50 µg/g creatinine was fond in the remaining districts (P < 0.01). Of the total study participants, the median concentration of Pb, Cd, and Cr was 19.48 µg/g creatinine, below the detection limit and 55.65 µg/g creatinine, respectively (Table 2).

**Table 2:** Pb, Cd and Cr distribution stratified by districts.

**List of districts**	**Pb** µg/g creatinine	**Cd** µg/g creatinine	**Cr** µg/g creatinine
BO−04	10.57 (6.48–24.4)	Bd (Bd-Bd)	69.18(41.49–109.14)
AD−04	10.43 (7.55–19.00)	Bd (Bd-Bd)	74.60 (43.73–158.48)
AK−07	20.23(8.24–35.33)	Bd (Bd−5.18)	13.63 (Bd −111.09)
AK −09	7.85 (Bd −15.88)	Bd (Bd −8.02)	8.58 (Bd −112.22)
AR−03	8.68 (Bd −14.35)	Bd (Bd-Bd)	45.00 (11.19–71.70)
BO−12	25.15 (15.00–42.74)	Bd (Bd-Bd)	50.07 (15.29–99.73)
GU−03	52.55(23.92–68.36)	0.52 (Bd −15.08)	76.60 (28.66–141.82)
GU−09	33.25 (16.54–61.33)	Bd (Bd-Bd)	51.79 (Bd −85.49)
KO−01	15.64 (Bd −36.03)	Bd (Bd-Bd)	70.38 (54.48–191.22)
KO−10	29.11 (13.90–43.48)	Bd (Bd−4.05)	81.61 (32.15–170.63)
KR−07	36.36 (16.66–48.42)	Bd (Bd−1.76)	24.84 (Bd −170.73)
KR−10	17.31 (11.06–41.00)	Bd (Bd-Bd)	88.49 (25.00–309.16)
LI−01	23.25 (10.67–46.83)	1.60 (Bd −11.98)	27.49(Bd −136.07)
NS−09	13.77 (10.20–22.50)	12.19 (Bd −20.40)	20.31 (6.88–102.39)
NS −11	19.72 (8.53–35.28)	Bd (Bd −8.65)	14.05 (Bd−43.58)
YK−02	28.90 (14.53–32.97)	Bd (Bd−6.93)	65.76 (36.06–350.68)
YK−12	33.34 (15.83–53.06	Bd (Bd−5.52)	122.61 (29.18–148.1)
Total	19.48 (9.45–38.84)	Bd (Bd−3.52)	55.65 (12.56–126.41)
P-value	0.00	0.00	0.00

Note: Bd refers below the detection limit (<0.001 µg/L)

**Fig. 2:**
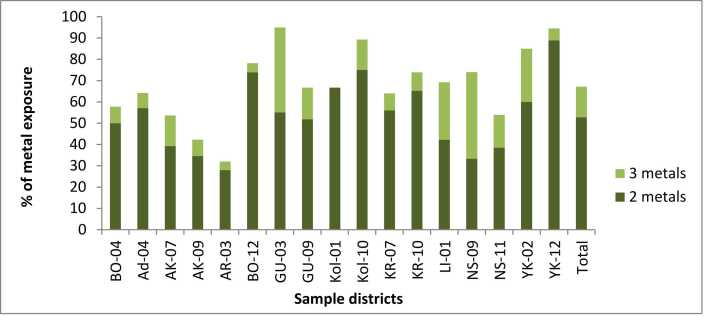
Multiple metal exposure in the residents by district.

The study also identified multiple metals in the residents of Addis Ababa. Three metals (Cd, Pb and Cr) were detected in 14.4 % of participants (Fig. 2). The distribution of these metals within each district indicates that, except for KO-01, between 4.0 % and 40.7 % of study participants had all three types of metals. The highest percentage of participants with triple metals exposure were found in NS-09 and Gu-03 districts at 40.7 % and 40 %, respectively. Moreover, over half of the total participants (52.8 %) had two types of metals. When analyzed by district, highest proportions of participants with two types of metals were observed in YK-12 and YK-02 districts, at 88.9 % and 60 %, respectively.

### 3.2 The distribution of heavy metals by socio-demographic characteristics

Urinary levels of Pb, Cd, and Cr were analyzed based on sex, BMI, age group, education level, occupation type and length of residence. Among the study participants, 67.6 % were females, 9.6 and 14.9 % were underweight (BMI < 18.5 kg/m^2^ and overweight (BMI ≥ 25 kg/m^2^) (Table 3). The age of participants ranged from 18 to 95, with 57.2 % falling within 18–35 years. About 16 % of participants had no formal schooling, while a higher proportion 32.1 and 30.9 % completed primary and secondary education, respectively. In terms of occupation, 44.4 % of participants were students or house wives, whereas, only 12 % work in industries and 6.5 % involved in solid waste management or street vending. About 49 % of participants lived in Addis Ababa for less than five years, while, the raining had resided there for five years or more.

**Table 3:** Urinary Pb, Cd, and Cr concentration based on socio-demographic characteristics (Mean value and range).

**Characteristics**	**N (%)**			**Pb µg/gcreatinine**			**Cd µg/gcreatinine**		**Cr µg/gcreatinine**
**Sex**									
Female	282 (67.6)			17.86 (7.71–35.86)			Bd (Bd−4.75)		59.44 (21.04–163.43)
Male	135 (32.4)			15.4 (7.74–34.09)			Bd (Bd- Bd)		59 (15.42–127.66)
P-values				0.002			0.014		0.807
**BMI (Kg/m2)**									
Underweight (<18.5)	40 (9.6)			23.53 (11.92–52.13)			Bd (Bd−3.47)		88.41 (28.1–183.27)
Normal (18.5–24.9)	315 (75.5)			19.34 (9.26–37.83)			Bd (Bd- 3.54)		48.44 (9.58–112.12)
Overweight (≥25)	62 (14.9)			20.29 (9.77–42.4)			Bd (Bd−1.46)		66.49 (34.3–148.97)
P-value				0.446			0.833		0.003
**Age (years)**									
18–35	237 (57.2)			19.84 (10.39–40.30)			Bd (Bd−4.43)		54.00 (13.42–116.60)
36–50	113 (27.3)			17.76 (8.35–33.42)			Bd (Bd- 2.7)		54.0 (9.24–129.40)
51–65	40 (9.7)			24.33 (8.48–52.81)			Bd (Bd−1.3)		78.96 (3.50–137.58)
> 66	24 (5.8)			20.29 (4.96–41.15)			Bd (B- Bd)		40.93 (21.01–233.73)
P-value				0.61			0.785		0.718
**Education level**									
No formal schooling	66 (15.8)			21.12 (10.28–39.13)			Bd (Bd- Bd)		72.5 (23.7–143.56)
Primary school	134 (32.1)			19.34 (9.63–41.69)			Bd (Bd- 3.21)		50.44 (10.5–131.5)
Secondary school	129 (30.9)			19.5 (9.95–35.27)			Bd (Bd−7.26)		57.87 (13.5–131.62)
College/university	88 (21.1)			17.2 (7.1–42.06)			Bd (Bd- 3.29)		48.77 (7.37–115.2)
P-value				0.855			0.382		0.567
**Occupation**									
Student & housewife	185 (44.4)			19.13 (10.49–35.08)			Bd (Bd- 4.83)		46.44 (10.9–109.93)
Industry	51 (12.2)			17.76 (6.71–29.82)			Bd (Bd- 4.32)		45.32 (9.8–122)
Solid waste & street vendors	27 (6.5)			19.67 (11.03–37.27)			Bd (Bd- 9.84)		78.08 (43.5–148.29)
Other	154 (36.9)			20.86 (8.88–42.91)			Bd (Bd- Bd)		67.32 (14.6–143.57)
P-value				0.565			0.16		0.105
**Resident year (years)**									
≤ 5	202 (48.4)			19.4 (9.79–37.49)			Bd (Bd- 3.25)		48.77 (8.69–114.86)
5.1–10	58 (13.9)			19.49 (7.05–44.67)			Bd (Bd−4.93)		78.28 (17–136.78)
> 10	157 (37.6)			19.62 (9.34–38.33)			Bd (Bd−3.39)		61.75 (20.6–142.62)
**Smoking**				0.983			0.403		0.102
Yes	12 (2.9)			18.65 (12.46–54.05)			Bd (Bd- Bd)		115.82 (1.46–193.89)
No	401 (97.1)			19.51 (9.42–38.48)			Bd (Bd- 3.54)		55.50 (12.78–121.12)
P -value				0.421			0.363		0.445
Total	417			19.48 (9.45–38.84)			Bd (Bd−3.52)		55.65 (12.6–126.41)

Note: Bd refers below the detection limit (<0.001 µg/L)

Pb concentration varied significantly between sex groups (p < 0.001), with female participants exhibiting a higher median concentration 17.86 µg/g creatinine compared to 15.40 µg/g creatinine in male participants. Among BMI groups, underweight participants had higher median Pb value of 23.53 µg/g creatinine than those who were normal weight or overweight, although this difference was not significant. There were no differences in Pb levels based on educational background, occupation type, and years of residency. The median Cd levels were below the detection limit across all socio-demographic characteristics. Still, a difference was observed between sex groups (P < 0.05) at the 75th percentile. Female participants had a Cd concentration of 4.8 µg/g creatinine at the 75th percentile while Cd was not detected in male participants. Cr was found in abundance in the urine sample of Addis Ababa residents with no difference in Cr concentration between female and male participants. Differences were noted among BMI groups; underweight and overweight participants had higher median Cr than normal-weight participants (P < 0.01). The median Cr was 88.41, 48.44, and 66.49 µg/g creatinine for underweight, normal-weight and overweight participants, respectively. Although there was no statistically significant difference, the median concentration of Cr was higher in the age group between 51 and 65 years. Participants who did not receive formal schooling had a higher median Cr (72.5 µg/g creatinine). In the occupation group, persons working in solid waste management and street workers had higher Cr (78.08 µg/g creatinine), compared to other occupation. The median concentration of Cr in smokers was higher than in non-smokers, measuring 115.82 and 55.50, respectively; however, this difference was not statistically significant.

### 3.3 Concentration of Pb, Cd, and Pb in tap water

The WHO guideline limit for heavy metals in drinking water is 10.0, 3.0 and 50.0 µg/L for Pb, Cd, and Cr, respectively. The mean concentration of Pb in drinking water samples was below the recommended limit in 7 districts (Table 4). However, a maximum concentration of Pb above the recommended limit was recorded in AD-04, AK-09 and GU-09 at 13.0, 15.0 and 15.0 µg/L, respectively. Cd was also not detected in tap water in 7 districts, while samples from the remaining 10 districts exceeded the WHO guideline limit. The highest concentration was found in KR-07, measuring 30 µg/L. Most water samples met the recommended limit for chromium (Cr); however, a maximum concentration of 50 µg/L was detected in sample NS-11, which is located near tannery industries in the district and upstream areas.

**Table 4:** Pb, Cd, and Cr concentration in drinking tap water.

LIST OF DISTRICTS	PB µG/L	CD µG/L	CR µG/L
BO−04	6.0 ± 5.0	7.5 ± 5.0	2.5 ± 2.8
AD−04	15 ± 7.07	Bd	15 ± 7.07
AK−07	Bd	Bd	3.3 ± 0.6
AK−09	13.3 ± 11.5	6.67 ± 4.7	6.7 ± 5.8
AR−03	Bd	Bd	15 ± 2.9
BO−12	5.7 ± 5.1	Bd	10 ± 0.0
GU−03	13.3 ± 5.8	6.67 ± 5.57	13.3 ± 5.8
GU−09	15.0 ± 7.0	Bd	25.3 ± 21.1
KK−01	Bd	5 ± 2.8	Bd
KK−10	Bd	6.67 ± 5.57	3.3 ± 3.0
KR−07	Bd	30 ± 0	20 ± 0
KR−10	5.0 ± 2.8	10.0 ± 0.0	40 ± 14.1
LI−01	10.0 ± 0.0	10 ± 0.0	10.0 ± 0.0
NS−09	4.0.0 ± 1.7	16.6 ± 5.8	26.7 ± 14.1
NS−11	5.0 ± 2.8	10 ± 0.0	50.0 ± 0.0
YK−02	Bd	Bd	Bd
YK−12	Bd	Bd	10.0 ± 7.0
WHO STANDARD	10	3	50

Note: Bd refers below the detection limit (<0.001 µg/L)

**Fig. 3:**
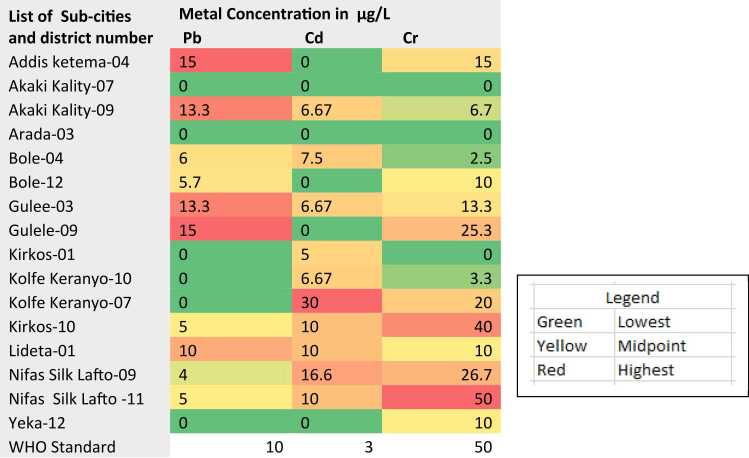
Area-wise heat map analysis of heavy metal contamination in Addis Ababa Sub-cities and selected districts.

The area-wise heat map analysis of heavy metal contamination in Addis Ababa Sub-cities revealed that Addis Ketema-04 has the highest Pb concentration at 15 µg/L, significantly exceeding the WHO standard of 10 µg/L (Fig. 3). Similarly, Kolfe K-07 demonstrating alarming Cd levels at 30 µg/L, followed by Nifas S-09 at 16.6 µg/L, and both Kirkos-10 and Lideta-01 at 10 µg/L, far above the WHO threshold of 3 µg/L, indicating a potential public health risk. Cr also emerged as a prevalent contaminate, with critical hotspots found in Kirkos-10 (40 µg/L) and Nifas S-11 (50 µg/L). Notably, industrialized areas such as Kolfie and Nifas Silk Sub-cities, exhibit the highest multi-metal pollution, particularly for Cr and Cd, likely linked to industrial activities, metalworking and garages. In contrast, residential areas such as Bole, Yeka Sub-cities show relatively lower contaminant levels, suggesting variations in exposure sources.

### 3.4 The distribution of metals by food consumption frequency

This study examined the differences in concentrations of metals (Pb, Cd and Cr) based on the consumption frequency of vegetable (such as tomatoes, kale, lettuce and cabbage), meat, milk, eggs and daily water intake habits (Table 5). In Addis Ababa, daily vegetable consumption is not widely practiced. Only 6.7 % of study participants consume vegetables every day, while the largest proportion, 50.4 %, consume them once a week. No significant difference in the concentration of Pb, Cd and Cr was found based on vegetable consumption frequency. Approximately 70 % of participants reported consuming meat once a month or less with 19.2 % consuming daily. The median concentration of Pb increased with more meat consumption (P-value < 0.05) reaching 25.57 µg/g creatinine for daily consumers, it was higher compared to those who consumed meat less frequently. The study also noted that 51 % of participants consumed milk daily but there was no difference in the levels of Pb, Cd and Cr based on milk consumption. Eggs were consumed daily by 37.7 % of participants. For those who consume eggs daily or two three times a week, Cd was detected at the 75th percentile measuring 10.87 and 23.57 µg/g creatinine, respectively. However, Cd was not detected in participants who consumed eggs less frequently. About 79 % of participants reported drinking two or more cups of water per day. Higher Cd concentrations (34.32 µg/g creatinine) were found in those who did not drink consistently or only drank when thirsty. In contrast, those who drank two or more cups of water daily had higher Cd levels at the 75th percentile (4.75 µg/g creatinine) (p < 0.00).

**Table 5:** Pb, Cd and Cr distribution by food consumption frequency and daily water consumption habit (mean value and range).

**Food consumption frequency**	**N (%)**	**Pb (µg/g creatinine)**			**Cd (µg/g creatinine)**					**Cr (µg/g creatinine)**
**Vegetables**										
**every day**	28 (6.7)		20.84 (9.63, 28.92)			Bd (Bd, 9.04)			34.89 (20.35, 131.34)	
**2–4 days a week**	53 (12.7)		18.78 (6.82, 42.16)			Bd (Bd, Bd)			49.09 (13.63, 119)	
**once a week**	210 (50.4)		18.63 (9.43, 36.99)			Bd (Bd, 5.93)			58.32 (12.3, 117.77)	
**once a month or less**	126 (30.2)		19.73 (10.58, 41.94)			Bd (Bd, Bd)			61.16 (10.81, 155.26)	
**P-Value**			0.96			0.069			0.681	
**Meat**										
**every day**	80 (19.2)		25.57 (13.71, 54.64)			Bd (Bd, 5.99)			82.54 (18.56, 138.42)	
**2–4 days a week**	10 (2.4)		16.78 (10.67, 35.45)			Bd (Bd, 0.72)			35.72 (Bd, 121.15)	
**once a week**	35 (8.4)		18.56 (10.38, 27.30)			Bd (Bd, Bd)			56.562 (6.89, 122.0)	
**once a month or less**	292 (70.0)		18.05 (8.07, 37.33)			Bd (Bd, 3.24)			52.10 (11.34, 114.48)	
**P-Value**			0.017			0.154			0.28	
**Milk**										
**every day**	214 (51.3)		19.29 (9.56,37.40			Bd (Bd, 4.63)			57.62 (14.19,136.20)	
**2–4 days a week**	28 (6.7)		15.08 (7.48, 47.61)			Bd (Bd, 2.63)			38.58 (7.78,126.88)	
**once a week**	61 (14.6)		18.78 (10.99, 35.08)			Bd (Bd, 8.47)			59.00 (19.48,95.85)	
**once a month or less**	114 (27.3)		21.00 (9.28, 40.17)			Bd (Bd, Bd)			52.85 (Bd, 134.34)	
**P-Value**			0.946			0.475			0.588	
**Egg**										
**every day**	157 (37.7)		19.67 (9.49, 44.27)			Bd (Bd, 10.84)			55.5 (12.02, 129.4)	
**2–4 days a week**	16 (3.8)		22.73 (6.51, 31.09)			Bd (Bd, 23.57)			31.09 (7.19, 112.14)	
**once a week**	85 (20.4)		15.66 (8.75, 31.43)			Bd (Bd, Bd)			60.51 (16.48, 121.12)	
**once a month or less**	159 (38.1)		19.45 (9.7, 39.71)			Bd (Bd, Bd)			52.1 (12.98, 134.11)	
**P-Value**			0.333			0.000			0.669	
**Glass of water/day**										
**two and more**	330 (79.1)		18.05 (8.89, 34.7)			Bd (Bd, 4.75)			55.18 (12.52, 128.94)	
**one to two**	81 (19.4)		23.69 (11.39, 52.59)			Bd (Bd, Bd)			57 (16.19, 110.84)	
**When they feel thirsty**	6 (1.5)		34.32 (29.5, 53.2)			Bd (Bd, Bd)			110.89 (Bd, 218.77)	
**P-Value**			0.006			0.038			0.869	

Note: Bd refers below the detection limit (<0.0001 µg/L)

### 3.5 Health risk assessment form tap water ingestion

The average concentration of heavy metals in the selected city water samples was followed the order: Cr (20.7 µg/L), Pb (9.12 µg/L) and Cd (8.52 µg/L) with peak concentration reaching 50 µg/L for Cr, 15 µg/L foe Pb and 30 µg/L for Cd. Although the concentrations are within the WHO permissible limits the cumulative effects pose significant health risks over a lifetime. The risk analysis revealed that the highest EDI for Pb was 0.00043 mg/kg/day observed in districts AD-04 and GU-09 (Table 6). The highest EDI for Cd is 0.00086 mg/kg/day observed in K-07 and the highest CDI for Cr is 0.0014 mg/kg/day observed in NS11 (Table 6). The highest. Hazard qu0tent (HQ) for Pb, is AD-04 and GU-09. Similarly the highest HQ for Cd and Cr was 0.31, 0.25 and 0.014 observed in KR-07 and NS-11 (Table 6). The highest HI is GU-03 (0.33) followed by GU-09 (0.31). Importantly, the HI of less than 1, suggested that no significant carcinogenic risk on from water consumption alone. The CILCR values vary significantly across districts, indicating different level of pollution and risks associated with heavy metals. The highest ILCR for Pb was observed in KR-10 at 1.22 × 10^−6^, for Cd in KR-10 at 1.75 × 10^−3^ and for Cr in NS-11 at 1.75 × 10^−3^. The highest CILCR values were found in districts such as KR-10 (1.9 ×10^−3^), LI-10 (2.33 ×10^−3^), and NS-11 (2.46 ×10^−3^) (Table 6), indicating a cancer risk from cumulative exposure to the assessed heavy metals.

**Table 6:** The estimated daily intake (EDI), Hazard quotient (HQ), Hazard Index (HI) and Cumulative Incremental Life Cancer Risk (CILCR) for the selected heavy metal exposure.

***Sample Districts***	***EDI (Pb)***	***EDI (Cd)***	***EDI (Cr)***	***HQ (Pb)***	***HQ (Cd)***	***HQ (Cr)***	***HI***	***ILCR (Pb)***	***ILCR (Cd)***	***ILCR (Cr)***	***CILCR***
*BO−04*	0.00017	0.00022	0.00007	0.122	0.061	0.0007	0.184	1.45 × 10⁻⁶	1.31 × 10⁻³	3.55 × 10⁻⁵	1.34 × 10⁻³
*AD−04*	0.00043	ND	0.00043	0.306	0	0.0043	0.31	3.65 × 10⁻⁶	ND	2.15 × 10⁻⁴	2.19 × 10⁻⁴
*AK−07*	ND	ND	0.00009	0	0	0.0009	0.00094	ND	ND	4.70 × 10⁻⁵	4.70 × 10⁻⁵
*AK−09*	0.0004	0.00019	0.00019	0.271	0.054	0.0019	0.327	3.23 × 10⁻⁶	1.16 × 10⁻³	9.50 × 10⁻⁵	1.26 × 10⁻³
*AR−03*	ND	ND	0.00043	ND	ND	0.00429	0.00429	ND	ND	2.15 × 10⁻⁴	2.15 × 10⁻⁴
*BO−12*	0.00016	ND	0.00029	0.116	ND	0.0029	0.119	1.39 × 10⁻⁶	ND	1.43 × 10⁻⁴	1.44 × 10⁻⁴
*GU−03*	0.00038	0.00019	0.00038	0.271	0.054	0.0038	0.329	3.23 × 10⁻⁶	1.16 × 10⁻³	1.90 × 10⁻⁴	1.36 × 10⁻³
*GU−09*	0.00043	ND	0.00072	0.306	0	0.00723	0.313	3.65 × 10⁻⁶	ND	3.62 × 10⁻⁴	3.66 × 10⁻⁴
*KK−01*	ND	0.00014	ND	ND	0.041	0	0.041	ND	8.72 × 10⁻⁴	ND	8.72 × 10⁻⁴
*KK−10*	ND	0.00019	0.00009	ND	0.054	0.00094	0.055	ND	1.16 × 10⁻³	4.70 × 10⁻⁵	1.21 × 10⁻³
*KR−07*	ND	0.00086	0.00057	ND	0.245	0.0057	0.251	ND	5.23 × 10⁻³	2.86 × 10⁻⁴	5.52 × 10⁻³
*KR−10*	0.00014	0.00029	0.00114	0.102	0.082	0.01143	0.196	1.22 × 10⁻⁶	1.75 × 10⁻³	5.71 × 10⁻⁴	2.33 × 10⁻³
*LI−01*	0.00029	0.00029	0.00029	0.204	0.082	0.00286	0.289	2.43 × 10⁻⁶	1.75 × 10⁻³	1.43 × 10⁻⁴	1.90 × 10⁻³
*NS−09*	0.00012	0.00047	0.00076	0.081	0.135	0.00763	0.224	9.69 × 10⁻⁷	2.89 × 10⁻³	3.82 × 10⁻⁴	3.27 × 10⁻³
*NS−11*	0.00014	0.00029	0.00143	0.102	0.082	0.0143	0.198	1.22 × 10⁻⁶	1.75 × 10⁻³	7.15 × 10⁻⁴	2.46 × 10⁻³
*YK−02*	ND	ND	ND	ND	ND	ND	ND	ND	ND	ND	ND
*YK−12*	ND	ND	0.00029	ND	ND	ND	ND	ND	ND	1.43 × 10⁻⁴	1.43 × 10⁻⁴

## 4 Discussions

In this study, the levels of Pb, Cd and Cr were measured in urine sample from the general population of Addis Ababa. A Mann-Whitney *U* test and kruskal-wallis H test were employed to evaluate the difference in metals concentrations across districts, socio-demographic characteristics, food consumption frequency and daily water intake habits. The result revealed that Pb was detected in urine from all districts with median concentration falling within the guideline limits set by to CDC. Nevertheless, higher concentrations exceeding these limits were found in the 75th percentiles of two districts. Female participants exhibited higher median concentration of Pb compared to male participants (p < 0.01), indicating that women are at a great risk of exposure to Pb than men. This finding aligns with the study of Anual et al. [8] and Eggers et al. [20]. It may be attributed the fact that women generally have lower creatinine levels than men, which can increase concentration of Pb in urine [16], [9]. Besides, men and women respond differently to chemical exposure, men having higher average body weight, organ blood flow, and plasma volume may eliminate chemicals from their bodies more efficiently than women [11]. Moreover, the decrease in bone mineral density after menopause may contribute to higher Pb exposure in women compared to men [62]. We also observed an increasing trend in Pb exposure levels among Addis Ababa residents over recent past years. This trend may be attributed to continuous industrial expansion, urbanization and poor waste management practice in the city [25], [60]. According to Central Statistics Agency of Ethiopia, CSA, (2001) and CSA, (2017) the number of large and medium scale manufacturing industries rose from 485 in 2000–1, 307 in 2016. Approximately, 70 % of waste is discharged into the environment without proper treatment, contaminating soil, air, rivers and vegetables grown in contaminated areas [49].

Studies by Jedrychowski et al. [32] and Santa Maria et al. [53] have reported that Pb exposure is linked to various neurological disorders including impaired cognitive function, Parkinson’s disease, and diabetes. Pb can easily transfer from a pregnant women to her fetus through the placenta, potentially affecting the central nervous system as reported by Vahter et al. [62]. A statistically significant positive difference was observed between meat consumption frequency and Pb level in urine as well as in water intake habits. Participants who drank two or more cups of water a day exhibited lower median concentrations of Pb compared to those who drank less amount of water a day. Although, no studies that directly connect meat consumption to Pb concentration, it is possible that the diet or the water consumed by animals may be contaminated by metals.

The median concentration of urinary Cd was below the detection limit in most districts, though, three districts had higher median values. Additionally, Cd levels exceeded 10 µg/g creatinine in the 75th percentiles of four districts. Key sources of human exposure to Cd includes smoking, drinking water and food [35]. In the current study, the impacts of smoking on urinary Cd could not be assessed as smoking is not prevalent in Addis Ababa. Studies by Shin et al. [58] indicate that drinking water and food are the major sources of Cd exposure in the general population if smoking is not the main factor. Drinking water and eggs consumption may contribute to Cd exposure. Participants who drank two or more cups of water a day exhibited higher Cd levels in the 75th percentile compared to those who drank less. As shown in (Table 4), drinking water contained significant level of Cd, Pb and Cr concentrations. Additionally, participants who consume eggs frequently had elevated levels of Cd in the 75th percentile compared to those who consume them less. Echeverría et al. [19] found that the concentration of Cd increased among individuals who consumed two eggs daily. Urinary Cd level reflects the body burden resulting from long-term exposure, highlighting the need for further research into other food sources as well. These findings are based on food consumption frequency data obtained from the questionnaire. It is recommended to analyze the heavy metal levels in various foods to gain a comprehensive understanding of the primary sources of exposure.

We did not find guideline values for creatinine adjusted Cr levels, leading us to compare our findings solely with those of Yard et al. [66] in Addis Ababa only. Cr was detected in all districts, with median urinary concentrations varying by district. The median Cr concentration across all districts appeared to have increased significantly tompared to Yard et al. [66]. We also observed variation in urinary Cr concentration among different BMI groups, with the highest Cr concentrations in underweight and overweight participants. Supporting our findings, Hong et al. [31] reported an increase in BMI correlating with higher Cr concentration in hair sample from Korean female adults. However, other studies did not find any association between BMI groups and Cr levels [14], [42].

For the general population, drinking water and food are the primary sources of non-occupationally exposure of Cr for general populations [58]. Although, we observed no difference in Cr concentrations associated with consumption of vegetables, eggs, meat, milk or daily water intake, other environmental sources such as occupation, contact with contaminated soil and industrial waste may contribute to overall exposure. Rowbotham et al. [50] reported that various industrial activities can lead to environmental contamination, posing risks to public health. In Addis Ababa, the presence of tanneries, paint production facilities and textiles that utilize Cr-containing chemicals raises concerns about potential exposure. The waste generated may not be fully removed by existing treatment systems, potentially contaminating soil, air, water, and vegetables which could be potential sources of exposure for the population [26]

Residents of Addis Ababa were found to be exposed to multiple metals, with 52.8 % of study participants exhibiting two types of metals and 14.4 % exhibiting three types in their urine samples. Such exposure can negatively impact the reproductive health. Gollenberg et al. [30] reported that exposure to Pb and Cd were found to be reproductive toxicants and contributing to delays in puberty in girls. This study placed significant emphasis on investigating the difference between metal exposure and vegetables consumption frequency, as a significant portion of vegetables grown in Addis Ababa are irrigated with water from the Akaki River, which has been found to be highly contaminated with heavy metals [2], [49], [64]. However, no significant difference was observed in vegetable consumption frequency concerning each metal separately and multiple metal levels in urine samples (P < 0.05). This lack of difference may be attributed to the infrequent consumption of vegetables among residents of Addis Ababa. More than 80 % of residents consume vegetables once a week or less. Furthermore, around 40 % of the vegetables consumed in Addis Ababa are produced near the Akaki River, while the remaining 60 % are sourced from other areas that may be free from metal contamination [49]. As a result, individuals eating vegetables from outside Addis Ababa may not be exposed to multiple metals.

Pb levels were generally higher in the 75th percentile of individuals among Gulele-03 and Gu-09. Additionally, the median Cd values of in Gu-03, LI-01 and NS-09 exceeded those of other districts. This may be due to the fact that these districts are located within in the Little Akaki River catchment, an area characterized by high population density, industries, including tannery industry, and both large and small enterprises [7]. According to Sung et al. [59], residents living near to industrial sites are often exposed to different pollutant, including heavy metals, through air, drinking water, food, and their environment.

The contamination of tap water with heavy metals suggests that residents of Addis Ababa may be at risk of exposure to these substances, potentially leading to bioaccumulation and adverse health effects over time. The Pb, CD and Cr EDI values were below oral reference dose (RfD) for Pb (0.0014), Cr (total) (0.1) and Cd (0.0035) mg/kg/day, respectively in most of the districts in agreement with the reports by [38] while [22] recorded high level CDI values. WHO (2017) reported that exposure to Pb through water is generally low as the main source of Pb in drinking water is old lead piping. However, peoples may expose to heavy metals with contaminated food including vegetables, meat and milk in urban and semi urban cities [24], [29]. High Pb concentration in the body can affect the Central nervous system, hematopoietic, cardiovascular, renal, gastrointestinal, endocrine, musculoskeletal, immunological, developmental and neurological systems (ATSDR, 2025; [6], [10], [48]). Higher Concentration of Cd also affects the liver, placenta kidney, brain, lungs and bone and cancer risks in human [24], [45], [46]. High concentration of Cr also causes kidney and liver toxicity and genotoxic carcinogens [45]. The analysis of ILCR across various districts reveals significant disparities in contamination levels. The highest ILCR for Pb was recorded in KR-10, which also had the greatest risk for Cd, indicating heightened health risks in certain areas. Districts such as NS-11 and LI-01, with elevated CILCR values, highlight the need for targeted environmental assessments and remediation strategies.

## 5 Conclusion

The median urinary concentration of Pb was within the CDC limit though highest concentration was observed in the 75th percentile among residents of two districts in Addis Ababa. Compared to the previous study, the median concentration from all samples shows an increase of more than tenfold. Female participants exhibited a higher median concentration of Pb than male participants. Difference between Pb levels and meat consumption frequency was noted with daily consumers showing higher median Pb levels than those who eat less frequently. Participants who drank two or more cups of water daily had lower Pb concentration. In terms of Cd, higher concentration above the ATSRD limit were found in the median values of three districts and in the 75th percentile of ten districts. Difference was also observed based on sex, egg consumption frequency and drinking water intake. Females, participants who frequently consumed eggs and those who drank two or more glass of water had higher Cd levels in the 75th percentile. In contrast, no difference was observed between Cr levels and the frequency of vegetables, eggs, meat or milk consumption as well as drinking water habits. In some districts, the tap water used for human consumption has been found to be contaminated with heavy metals. This suggests that residents of Addis Ababa may be at risk of exposure to these metals, which can lead to bioaccumulation and adverse health effects over time. Assessments of the incremental lifetime cancer risk (ILCR) associated with heavy metals indicate a heightened cancer risk in certain areas, indicating the need for targeted environmental assessments and remediation strategies. To address these concerns, it is crucial to implement systematic monitoring of drinking water sources and to conduct biomonitoring of heavy metal exposure and its trends.

## Authors contributions

TAA wrote the first draft and performed formal analysis and interpretation with supervision from AMH and TAT. AMH contributed proposal write up to data analysis. TAT rewrite the manuscript for publication. All Co-authors reviewed, edited and contributed to the revision of the final draft and finance the research.

## CRediT authorship contribution statement

**Tsigereda Assefa Alemayehu:** Writing – original draft, Visualization, Software, Methodology, Investigation, Formal analysis, Data curation, Conceptualization. **Tadesse Alemu Terfie:** Writing – review & editing, Supervision, Methodology, Formal analysis, Data curation. **Mehari Meles:** Software, Methodology, Investigation, Data curation. **Andualem Mekonnen Hiruy:** Writing – review & editing, Supervision, Methodology, Funding acquisition, Formal analysis, Data curation, Conceptualization. **Belay Tefera:** Software, Resources, Methodology, Conceptualization.

## Ethical clearance

The ethical approval protocol was approved by the Ethiopian Public Health Institute scientific and Ethical review board.

## Funding

Addis Ababa University, research directorate, Thematic research award No. TR/001/2021.

## Declaration of Competing Interest

The authors declare that they have no known competing financial interests or personal relationships that could have appeared to influence the work reported in this paper.

## Data Availability

I confirm that all data related to this manuscript is included within the article. Therefore, no additional data or code needs to be shared separately.
